# A role for phosphodiesterase type 5 inhibitors in remodelling the urinary bladder after radiation exposure

**DOI:** 10.1371/journal.pone.0242006

**Published:** 2020-11-09

**Authors:** Hee Youn Kim, Dong Sup Lee

**Affiliations:** Department of Urology, St. Vincent’s Hospital, College of Medicine, The Catholic University of Korea, Seoul, Republic of Korea; National Institutes of Health, UNITED STATES

## Abstract

Minimizing the toxicity of radiotherapy is challenging. We investigated the effects of a phosphodiesterase type-5 inhibitor (PDE5I) on the urinary bladder after pelvic radiotherapy. Eight rats were assigned to each group (group 1: control; group 2: radiation; group 3: radiation plus PDE5I). Radiation dose was 10 Gy/one fraction. Udenafil (20 mg/kg, daily for 4 weeks) was administered in group 3. Cystometry was performed 4 weeks after treatment, followed by real-time PCR for PDE5, vascular endothelial growth factor (VEGF), and endothelial nitric oxide synthase (eNOS) mRNA, western blotting for PDE5, cyclic GMP-dependent protein kinase (PRKG), VEGF_164_, Akt, eNOS and NADPH oxidase (NOX)-2 proteins, and immunohistochemistry for eNOS. The expression of both VEGF mRNA and eNOS mRNA was higher in group 3 than in group 2. VEGF and eNOS protein expression improved with PDE5I treatment. Akt protein phosphorylation was higher in group 3 than in group 2, but NOX-2 protein expression was lower in group 3 than in group 2. Immunohistochemistry showed that the mean density of arterioles expressing eNOS was higher in group 3 than in group 2. Cystometry revealed that the intercontraction interval was remarkably longer in group 3 than in group 2 but that the maximal voiding pressure was higher in group 2 than in group 3. Daily treatment with a PDE5I after radiotherapy may prevent bladder storage dysfunction, potentially due to its effects on vasodilation and angiogenesis and through minimizing tissue oxidative damage by means of the VEGF/Akt/eNOS pathway.

## Introduction

Radiotherapy is essential for the nonsurgical curative treatment of cancer. However, in the case of pelvic radiotherapy, a decrease in urinary function due to the toxicity of radiotherapy should be considered [1]. Over 20 years, hypofractionated external beam radiotherapy has been used to treat prostate cancer [2]; however, urinary toxicity remains a bothersome complication after radiotherapy [3]. Radiotherapy-induced genitourinary adverse effects are scaled from 0 to 5 according to the Radiation Therapy Oncology Group (RTOG) criteria [4], including symptoms and signs such as frequency, dysuria, haematuria, and telangiectasia. In a previous study investigating the effect of radiotherapy on localized prostate cancer with over 5 years of follow-up, less than 3% of cases had an RTOG grade of 3 or more; however, acute low-grade (grade 1 or 2) genitourinary morbidity was over 80%, and late low-grade morbidity was over 60% regardless of radiation dosage [5]. Underlying the pathophysiology of radiation-induced cystitis includes progressive endarteritis that leads to poor tissue oxygenation, for which hyperbaric oxygenation is indicated to treat bladder complications [6].

Meanwhile, phosphodiesterase type-5 inhibitors (PDE5Is) have been a first-line treatment option for erectile dysfunction. Among the beneficial actions of PDE5Is on tissues, neovascularization is expected to contribute to tissue survival by minimizing tissue damage to stress [7]. Nomiya et al. suggested that PDE5Is could be prophylactic drugs for bladder dysfunction by preventing chronic bladder ischaemia [8]. However, there is a lack of evidence for the effect of PDE5Is on urinary bladder function after pelvic radiation therapy. Therefore, we aimed to identify how PDE5Is affect the urinary bladder after pelvic irradiation in an animal model. The primary objective of the study was to observe molecular changes, including endothelial nitric oxide synthase (eNOS) activity and relevant protein activity, and the secondary objective was to examine the cystometric effects of PDE5Is. We hypothesized that eNOS and relevant protein activity would deteriorate after radiotherapy and that PDE5I treatment could ameliorate hypoxic tissue damage via an eNOS-related molecular pathway.

## Materials and methods

### Ethical approval

All experimental protocols were approved by the Institutional Animal Care and Use Committee at St. Vincent’s Hospital, The Catholic University of Korea (approval no. 17–01; date: Jan 26, 2017). All experiments were performed in the Laboratory at St. Vincent’s Hospital under standard conditions. The experiment adhered to the Animal Research: Reporting of *In Vivo* Experiments (ARRIVE) guidelines [9]. All surgery was performed under sodium pentobarbital anesthesia, and all efforts were made to minimize suffering.

### Experimental setting preparation

**Fig 1:**
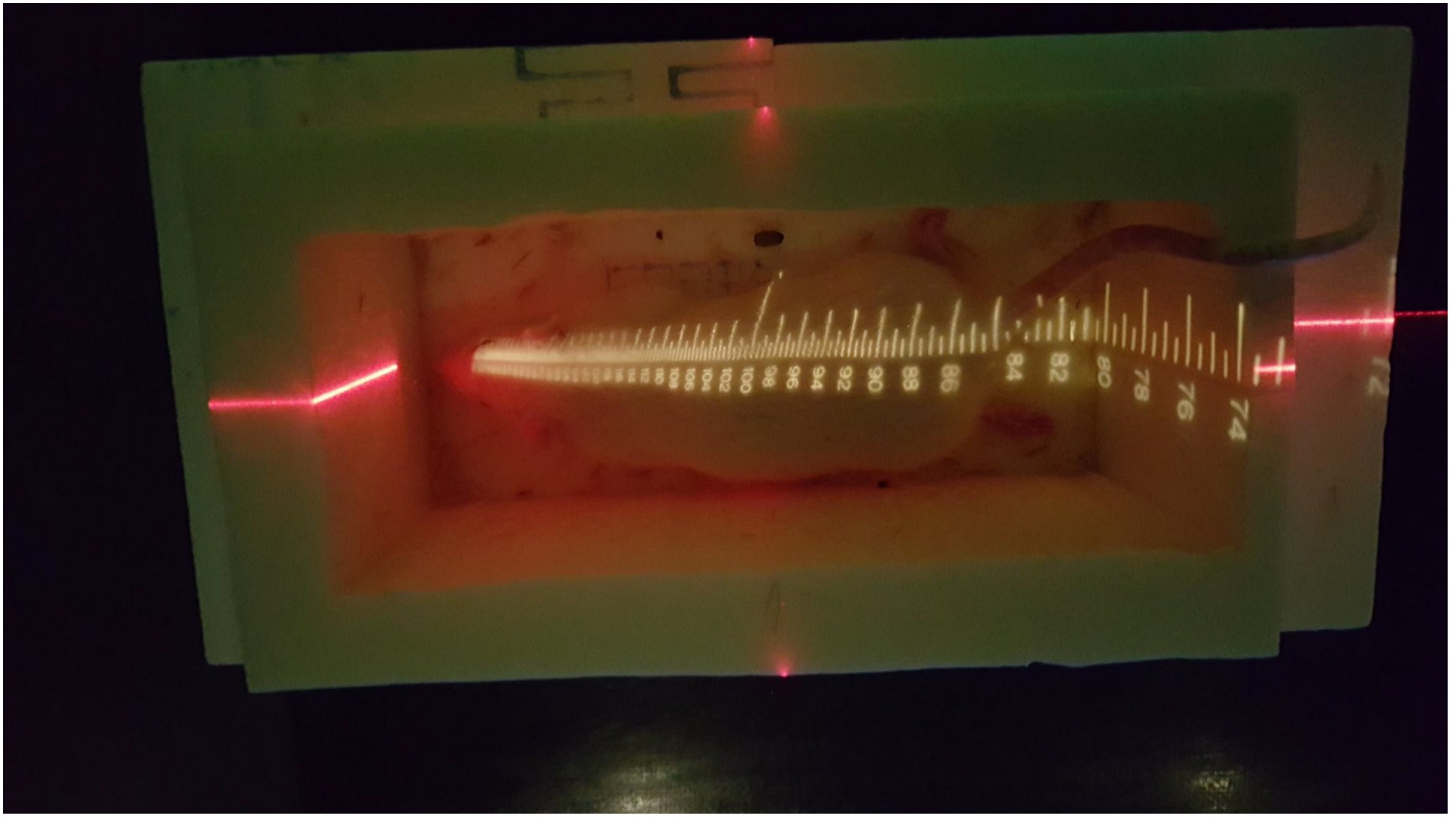
Preparation for radiation treatment. Before radiation treatment, a rat was anaesthetized in a plastic cage of the appropriate size. After simulation using computed tomography, the alignment was confirmed using laser beam as shown in the figure. The irradiation source was 6 MV photon without filter, and dose rate was 300 monitor unit per minute.

Twenty-four male Wistar rats (14~15 weeks old, 300~350 g, KOATECK, Pyeongtaek, Korea) were used. The animals were housed in standard polypropylene cages in a temperature-controlled room (25°C ± 1°C) with a 12:12-h light/dark cycle (lights on at 7 AM) and were allowed free access to food and water for 1 week of acclimation. Eight rats were assigned to each group (group 1 as the control group, group 2 as the radiation group, and group 3 as the radiation plus PDE5I group). We checked animal well-being state twice in a day (during feeding animals and cleaning of cages at am 8:00, and during medication via nasogastric tube at pm 1:00). The recommended radiation dosage for prostate cancer (for both primary treatment and salvage treatment) is 70~80 Gy [10]. To translate this dosage to the experimental setting, we used the biological effective dose and alpha-to-beta ratio. We intended one fraction exposure, and the alpha-to-beta ratio for prostate cancer is known to be 1.5 [11]; therefore, the biological effective dose can calculated as ‘d^2^/1.5+d’ (d means dose per fraction). Using this logic, we applied 10.0 Gy/one fraction (biological effective dose of 76.7 Gy) to the prostate and urinary bladder in groups 2 and 3. Before pelvic irradiation, 30 mg/kg pentobarbital was administered via intraperitoneal injection. Each anaesthetized rat was placed in a plastic enclosure made to the size of the rats (Fig 1). Then, radiation was targeted to the prostate and bladder. Rats in group 1 were anaesthetized but not exposed to radiotherapy.

In group 3, 20 mg/kg udenafil (Zydena, Dong-A, Seoul, Korea) was administered daily via nasogastric tube for 4 weeks beginning one day after radiation treatment. Instead of udenafil, saline was administered daily for 4 weeks in groups 1 and 2.

### Cystometry

Four weeks after radiation exposure, all rats were anaesthetized with intraperitoneal pentobarbital (30 mg/kg). A 24-gauge needle connected to a polyethylene catheter was inserted into the dome of the urinary bladder after laparotomy. Saline was continuously infused (0.1 ml/min) into the bladder with a KD Scientific syringe pump (KD Scientific Inc., Holliston, MA, USA), and the intravesical pressure was checked with a pressure transducer (Harvard Apparatus, Holliston, MA, USA). The intercontraction interval (ICI), maximum voiding pressure (MVP), and baseline pressure were measured.

### Immunohistochemistry (IHC)

After cystometry, bladder tissues were obtained for IHC, RNA extraction, and western blotting. Then all rats were sacrificed using CO_2_ inhalation. The urinary bladder was equally divided by sagittal incision after total cystectomy; half of the bladder was cryoprotected for measurements of mRNA and protein expression, and the other half of the bladder was immediately fixed with 10% formalin. Fixed tissues were embedded in paraffin, cut into 4-μm-thick sections, and placed on glass slides treated with poly-L-lysine. The specimens were incubated at 58°C, deparaffinized with xylene, and sequentially rehydrated in 100%, 90%, 80%, and 70% alcohol solutions. To evaluate endothelial function and discriminate the vascular structure from the surrounding structures, we used a primary antibody targeting endothelial nitric oxide synthase (eNOS; 1:100; Thermo Fisher Scientific, Waltham, MA, USA). Heat-induced antigen retrieval was applied, where tissues were boiled in a citrate buffer solution by heating in a microwave for 2 min [12]. The ImmPress kit (Vector Laboratories, Burlingame, CA, USA) was used for secondary antibodies where Tris-buffered saline was used to wash out the specimen. A 3,3-diaminobenzidine tetrahydrochloride (DAB) kit was used for visualisation. Three sections from each rat were analysed. Three sections from each rat were randomly chosen and analyzed using 400x magnification by a pathologist who was blinded to the groups. Therefore, 24 samples for each group and total 72 samples were investigated. A semiquantitative evaluation of eNOS expression was performed by measuring staining intensity (0, 1+, 2+, 3+, or 4+) in a fixed field. A semiquantitative evaluation for submucosal venous engorgement (0, 1+, 2+, 3+, or 4+) was also performed in a fixed field: no venule as ‘0’; venules having comparable size with adjacent arterioles as ‘1+’; 1~2 venules larger than adjacent arterioles as ‘2+’; 3~4 venules larger than adjacent arterioles as ‘3+’; numerous venules larger than adjacent arterioles as ‘4+’.

### RNA extraction

RNA was extracted using TRIzol solution (Ambion; Thermo Fischer Scientific), and RT-PCR was performed using the conditions described in the S1 Table. We used the glyceraldehyde-3-phosphate dehydrogenase (GAPDH) gene as a housekeeping gene (National Center for Biotechnology Information accession no. NG 028301).

### Western blot analysis of protein expression

The procedure used for western blotting was similar to the method reported in a previously published study [13]. A portion of each bladder was frozen and homogenized in protein extraction buffer (Intron, Seongnam, Korea) containing protease inhibitors (Sigma, MO, USA). The homogenates were centrifuged at 12,000 rpm for 20 min. The total protein concentration in the supernatant was measured using the Bradford assay (Bio-Rad, CA, USA). Equal amounts of protein (10 μg) were electrophoretically separated on 10% SDS-PAGE gels and transferred to a PVDF membrane (Bio-Rad, CA, USA). The membrane was blocked with 5% skim milk (Bio-Rad, CA, USA) containing 0.1% Tween 20 for 1 h and incubated with antibodies against PDE5 (1:1000, Abcam, Cambridge, UK), cyclic GMP-dependent protein kinase (1:1000, PRKG; LifeSpan BioSciences, WA, USA), vascular endothelial growth factor 164 (VEGF_164_; 1:1000, Abcam, Cambridge, UK), Akt (1:1000, Cell Signaling, Massachusetts, USA), phosphorylated (*p*-) Akt (1:1000, Cell Signaling, Massachusetts, USA), eNOS (1:1000, Abcam, Cambridge, UK), and β-actin (1:1000, Abcam, Cambridge, UK) overnight. The secondary antibody was incubated with the membrane at room temperature for 1 hour. The expression of each protein was finally confirmed using enhanced chemiluminescence (ECL; Bio-Rad, CA, USA). The results of the ECL reaction were measured using a LAS-4000 mini luminescent image analyser (GE Healthcare, IL, USA). The results were quantified using densitometry.

### Statistical analysis

SPSS (IBM Corp. Released 2012. IBM SPSS Statistics for Windows, Version 21.0. Armonk, NY: IBM Corp.) was used for the statistical analysis. The number of experimental animal needed (total twenty-four, eight for each group) was determined based on the ‘resource equation’ approach because of the exploratory nature of the current study [14]. The Kruskal-Wallis test was performed to compare the 3 groups, and the Mann-Whitney test with Bonferroni’s correction was performed as the post hoc test. Statistically analysed data are presented as the means ± standard errors. Values of *p* < 0.05 are considered statistically significant.

## Results

### mRNA expression

**Fig 2:**
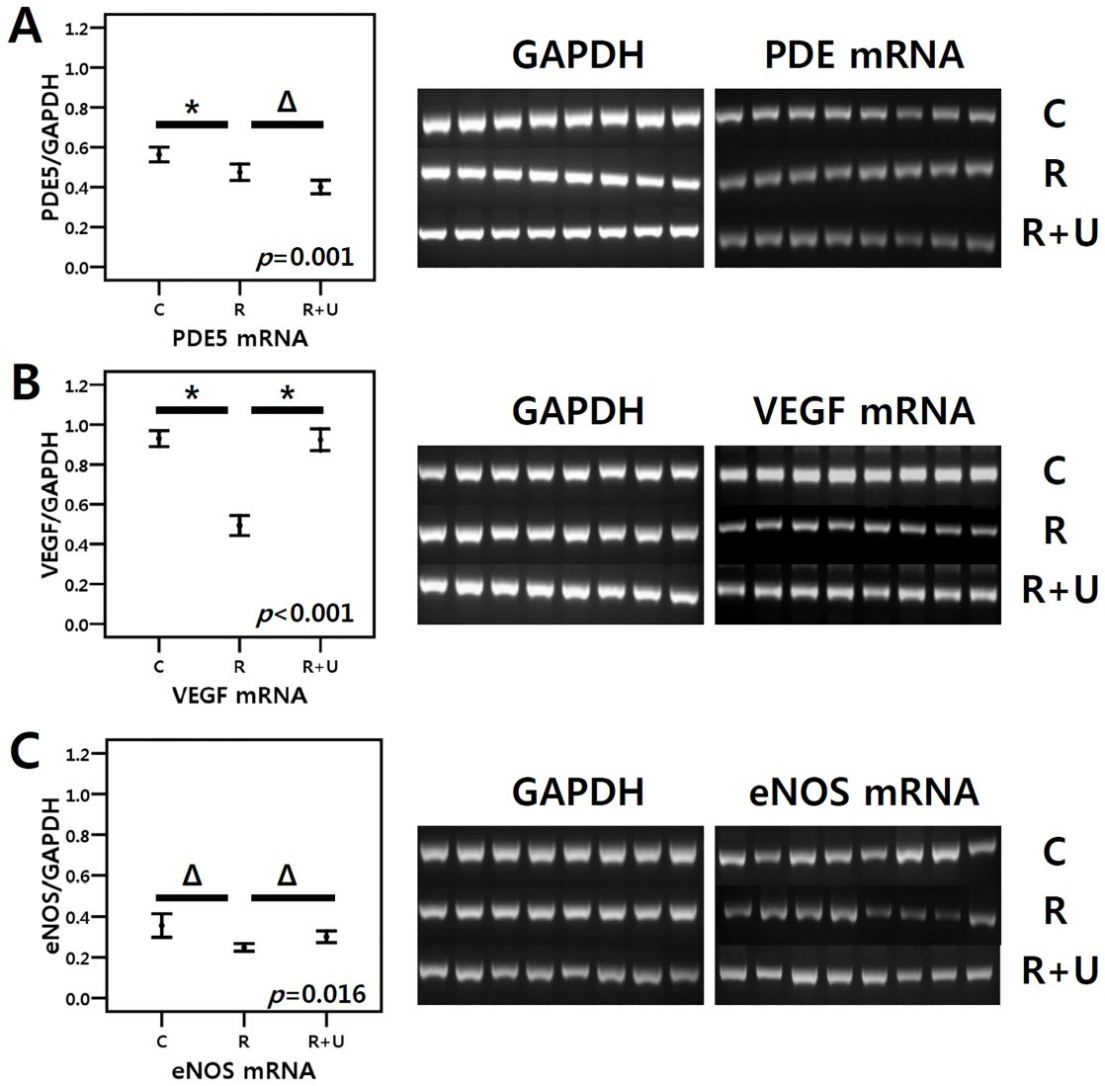
RT-PCR for evaluating mRNA expression in the urinary bladder. Daily PDE5I administration further decreased PDE5 mRNA expression (*p* = 0.084, group 2 vs. group 3), although the difference did not reach statistical significance (A). Daily PDE5I administration ameliorated the decrease in VEGF mRNA expression (B) and it tended to lessen the decrease in eNOS mRNA expression (C). The *p*-value in each box was calculated by the Kruskal-Wallis test to compare the three groups. The asterisk (*) indicates *p*<0.05 after the post hoc test; the triangle symbol (Δ) indicates 0.05 < *p* < .10 after the post hoc test. C (group 1): control, R (group 2): radiation, R+U (group 3): radiation plus udenafil.

The mean PDE5, VEGF and eNOS mRNA expression differed among the three groups (*p* = 0.001, *p*<0.001 and *p* = 0.016, respectively) (Fig 2A). PDE5 mRNA expression tended to be lower in the group administered PDE5I for 4 weeks after radiation (group 3) than in radiation treatment groups (group 2 vs. group 3, *p* = 0.084 after post hoc test). Radiation treatment significantly reduced the expression of VEGF mRNA expression; however, VEGF mRNA expression was maintained when daily PDE5I treatment was applied (Fig 2B). The radiation treatment group (group 2) showed a trend towards lower eNOS mRNA expression than the control group (group 1 vs group 2, *p* = 0.063 after post hoc test), but daily PDE5I treatment lessened this decrease (group 2 vs. group 3, *p* = 0.063 after post hoc test) (Fig 2C).

### Western blot analysis

**Fig 3:**
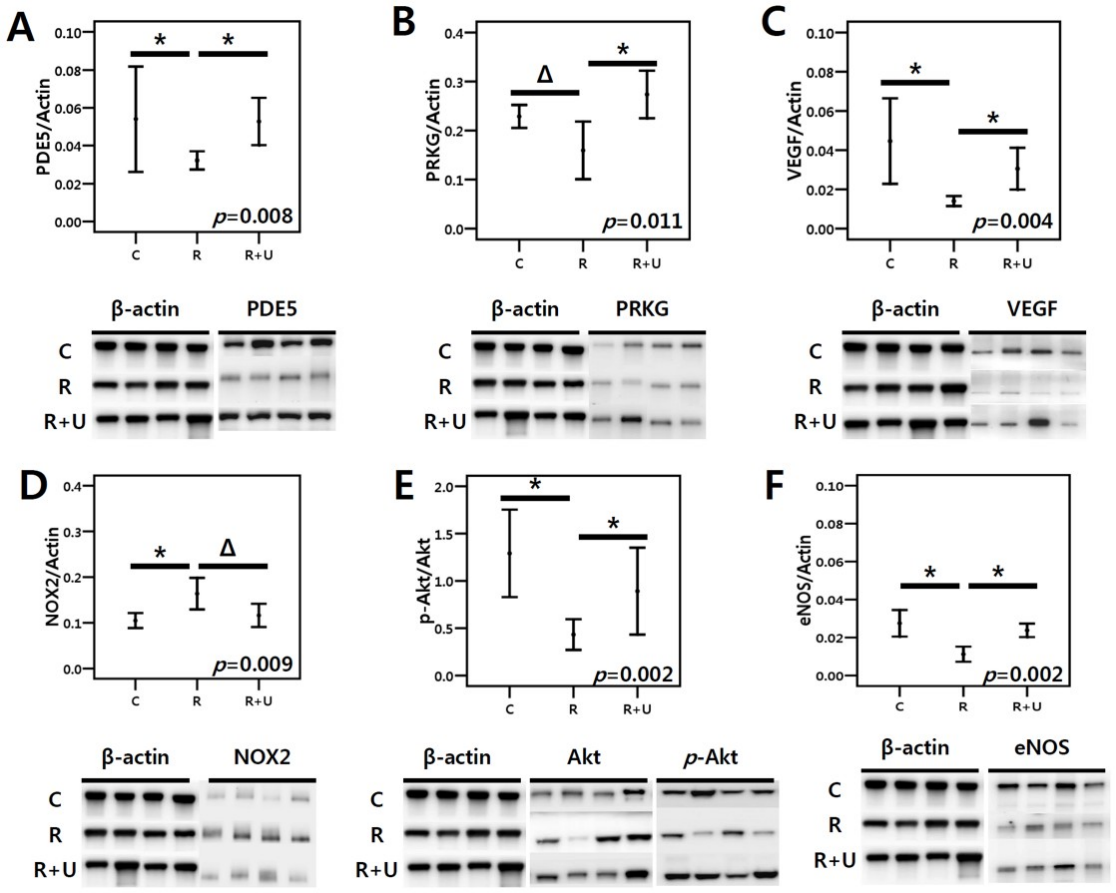
Western blotting analysis of PDE5, PRKG, VEGF, NOX2, Akt, and eNOS protein expression. Daily PDE5I administration after radiation therapy ameliorated the decrease in PDE5 (A), PRKG (B), VEGF (C), p-Akt to Akt ratio (E), and eNOS (F). PDE5I administration tended to lessen the level of NOX-2 after radiation therapy (D). The *p*-value at the top of each box was calculated by the Kruskal-Wallis test to compare the three groups. The asterisk (*) indicates *p* < 0.05 after the post hoc test; the triangle symbol (Δ) indicates 0.05 < *p* < 0.10 after the post hoc test. C (group 1): control, R (group 2): radiation, R+U (group 3): radiation plus udenafil.

The mean expression of PDE5 protein was lower in the radiation treatment group (group 2) than in the control group (group 1), but continuous PDE5I treatment elevated the mean expression of PDE5 (group 2 vs. group 3, *p* = 0.015 after post hoc test) (Fig 3A). PRKG expression also tended to be lower in the radiation treatment group (group 2) than in the control group (group 1); however, daily administration of the PDE5I significantly increased PRKG expression (group 2 vs. group 3, *p* = 0.021 after post hoc test; Fig 3B). Furthermore, radiation exposure significantly reduced VEGF protein expression, but PDE5I treatment reversed the effects of radiation on VEGF protein expression (group 2 vs. group 3, *p* = 0.030 after post hoc test; Fig 3B) (Fig 3C). NOX-2 levels were higher in the radiation group (group 2) than in the control group (group 1) but tended to decrease after PDE5I treatment (*p* = 0.063 after post hoc test). (Fig 3D). The mean ratio of *p-*Akt to total Akt expression was significantly higher in the group treated with the PDE5I (group 3) than in the radiation treatment group (group 2) (group 2 vs. group 3, *p* = 0.015 after post hoc test) (Fig 3E). Finally, eNOS expression was also higher in group 3 than in group 2 (*p* = 0.001 after post hoc test) (Fig 3F).

### Histology and IHC

**Fig 4:**
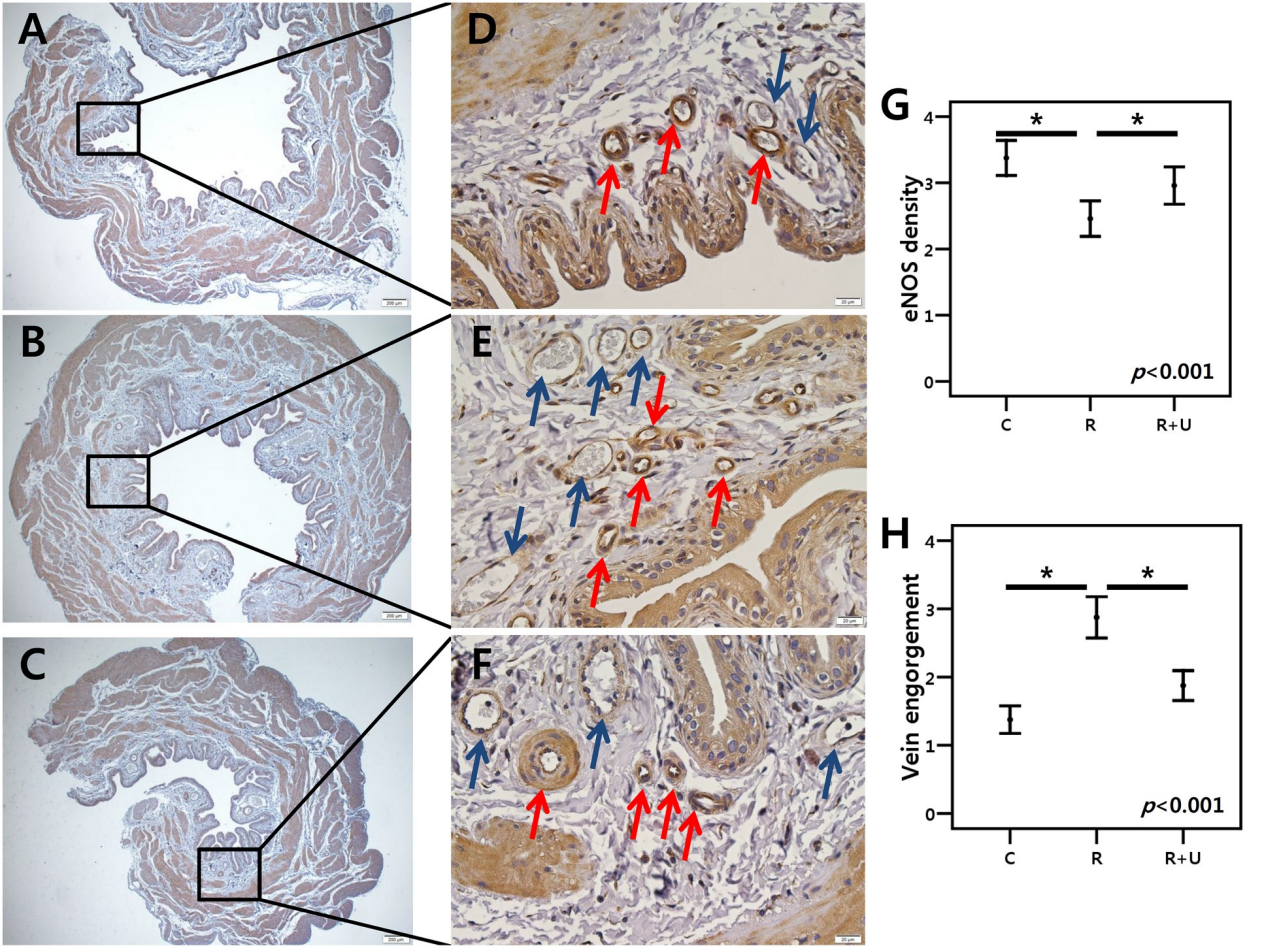
IHC for eNOS and overall histology. A, B, and C show group 1, group 2, and group 3, respectively, at 40x magnification (A-C). Scale bar at the corner of A-C: 200 μm. Arteries in each group were further magnified within the box (D-F). Scale bar at the corner of D-F: 20 μm. In group 2 (E), a thick oedematous submucosal layer and many engorged veins (blue arrow) were observed. PDE5I administration ameliorated the decrease in eNOS staining density of arterioles (red arrow) (G), as well as the increase in submucosal venous engorgement (H). The *p*-value in each box was calculated by the Kruskal-Wallis test to compare the three groups. The asterisk (*) indicates *p* < 0.05 after the post hoc test. Blue arrow: venule, Red arrow: arteriole. C (group 1): control, R (group 2): radiation, R+U (group 3): radiation plus udenafil.

At a glance (Fig 4A–4C, x40), we found differences in submucosal thickness among the three groups. Upon higher magnification (Fig 4D–4F, x400), the mean density of eNOS in the submucosal arterioles was lower in group 2 than in groups 1 and 3. Semiquantitative analysis revealed that eNOS density was the lowest in group 2 (*p*<0.001) and that eNOS density higher in group 3 than in group 2 (*p* = 0.008 after post hoc test) (Fig 4G). Compared to the control group, groups 2 and 3 more frequently exhibited engorged submucosal veins, but this engorgement was more noticeable in group 2 than in group 3 (Fig 4D–4F and 4H).

### Cystometry

**Fig 5:**
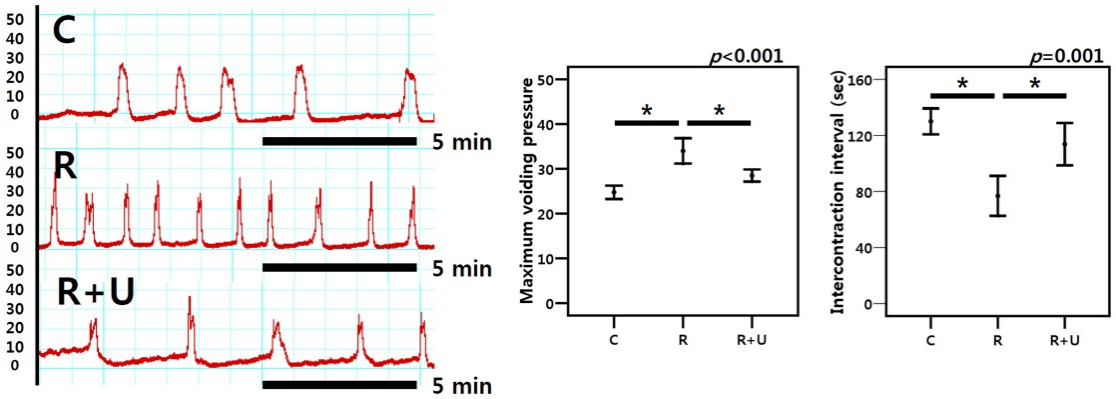
Cystometry. The ICI was shorter in group 2 than in group 1 but was improved with PDE5I administration. MVP was the highest in group 2, but PDE5I administration ameliorated the pressure. The *p*-value at the top of each box was calculated by the Kruskal-Wallis test. The asterisk (*) indicates *p* < 0.05 after the post hoc test. C (group 1): control, R (group 2): radiation, R+U (group 3): radiation plus udenafil.

The mean duration of the ICI in groups 1, 2, and 3 was 130.0 s, 76.9 s, and 113.8 s, respectively (*p* = 0.001; Fig 5), revealing that daily PDE5I treatment elongated the ICI after radiation therapy (*p* = 0.005 after post hoc test). Finally, the MVP was the highest in group 2 but was reduced by PDE5I treatment (*p* = 0.015 after post hoc test).

## Discussion

PDE5 is expressed in many tissues, including the corpus cavernosum, urinary bladder, and prostate [15]. PDE5 breaks cyclic guanosine monophosphate (cGMP); therefore, PDE5Is block the degradative action of the cGMP-specific PDE5 in the smooth muscle cells lining the vessels supplying relevant tissues [16], thereby prolonging the action of cGMP and relaxing the smooth vasculature. Moreover, previous studies have shown that PDE5Is activate eNOS and generate nitric oxide (NO) [17, 18]. NO regulates the degree of vascular smooth muscle cell contraction, mainly by stimulating soluble guanylyl cyclase () to produce cGMP. Vasodilation, a beneficial action of PDE5Is, is therefore a focus of research on the function of PDE5Is. The activated NOS pathway not only contributes to vasodilation but also maintains adenosine triphosphate (ATP) synthase and calcium homeostasis via the PRKG pathway, which eventually contributes to cell survival [19]. In addition, many studies have demonstrated that PDE5Is stimulate angiogenesis by upregulating VEGF expression [20].

As described previously, endarteritis and decreased tissue oxygenation are key pathophysiological features of radiation toxicity that increase superoxide formation, resulting in tissue inflammation and fibrosis [6, 21]. A previous animal study emphasized that nicotinamide adenine dinucleotide phosphate (NADPH) oxidase activation is closely associated with radiation toxicity [22].

Based on the aforementioned evidence, we investigated the effects of a PDE5I on the urinary bladder after radiation exposure. Despite radiation lowering the expression of PDE5 mRNA in the present study, chronic PDE5I treatment caused an accumulation of PDE5 protein, resulting in lower PDE5 mRNA expression in group 3 than in group 2. In contrast, PRKG expression was significantly higher in group 3 than in group 2.

VEGF mRNA expression was greatly upregulated in response to PDE5I treatment. As a result, VEGF protein expression in radiation-exposed bladder tissue was significantly higher in the group given PDE5I treatment. Among the various isoforms of VEGF, we examined VEGF_164_. In the myocardium, the absence of VEGF_164_ impaired myocardial angiogenesis, leading to ischaemic cardiomyopathy [23]. Furthermore, VEGF_164_ is essential to glomerular angiogenesis and renal arteriogenesis in postnatal mice [24]. However, VEGF expression is not always favourable. In a rat model of cyclophosphamide-induced cystitis, VEGF_164_ seems to be overexpressed [25]; thus, several researchers have investigated the effects of anti-VEGF treatment on bladder pain and voiding function in a rat model of cyclophosphamide-induced cystitis. Lai et al. emphasized that systemic anti-VEGF treatment was not effective in normalizing the increased urinary frequency or small voided volumes that developed after cyclophosphamide-induced cystitis but may be effective for pelvic pain [26]. In another study investigating the effect of low-intensity shock wave therapy on erectile dysfunction, the authors concluded that upregulated VEGF and eNOS expression were key therapeutic mechanisms (angiogenesis) for enhancing erectile function [27]. Therefore, although isoforms of VEGF may act differently in different circumstances, VEGF_164_ is associated with angiogenesis and may be involved in tissue remodelling. These issues regarding the effect of VEGF expression on the urinary bladder require further investigation. In the present study, radiation treatment of the urinary bladder decreased VEGF_164_ expression at 4 weeks after radiation exposure, but daily PDE5I treatment upregulated VEGF_164_ expression.

In the present study, PDE5I treatment tended to upregulate eNOS mRNA expression. Both IHC and western blotting revealed that eNOS protein expression was significantly increased with PDE5I treatment (Figs 2C, 3F and 4). As mentioned before, activation of eNOS releases NO, which is essential to PRKG activation. However, superoxide free radicals decrease NO bioavailability [28]. In the present study, NOX-2 (gp91-PHOX, an isoform of NADPH oxidase that is known to participate in an enzyme complex generating superoxide anion) was significantly elevated after radiation exposure, but PDE5I treatment ameliorated the increase in its expression. PDE5I treatment has been shown to have the same effect on superoxide levels in erectile tissue in the radiation setting [22]. The Akt signalling pathway is also crucial to this discussion. Akt, named protein kinase B, can be activated by VEGF in endothelial cells, and phosphorylation of Akt activates eNOS, through which NO is released, contributing to vasodilation [29]. Thus, the Akt pathway is strongly entangled with the eNOS and VEGF pathways.

Thickening of the lamina propria and telangiectasia are typical histologic findings in radiation-induced cysts [21]. An obvious submucosal thickening was observed after radiation therapy in the present study (Fig 4), where numerous engorged veins were identified in the radiation-exposed tissue. Daily PDE5I treatment lessened this thickening and engorgement. A previous study demonstrated that a PDE5I was effective at preserving urinary bladder function after chronic ischaemia [8]. Similarly, PDE5Is may be able to rescue bladder tissue from radiation toxicity by remodelling the bladder by means of vasodilation and angiogenesis, thereby preserving bladder compliance and/or capacity. Indeed, the cystometry results of the present study provide support for this possibility.

The authors want to note the limitations of this study. Due to animal ethical issues, only a minimal number of animals (8 in each group) were sacrificed. Therefore, the standard errors were relatively large in several items, which might lead to a post hoc *p*-value higher than 0.05. Furthermore, the inclusion of another group treated with a different signalling inhibitor, such as a VEGF inhibitor, may further our understanding of the signalling connection among VEGF, Akt, and eNOS. Although eNOS has been known as a bio-marker responding to PDE5I, the concomitant use of phosphorylated eNOS with eNOS during western blot in the future study could show clearer results.

## Conclusions

**Fig 6:**
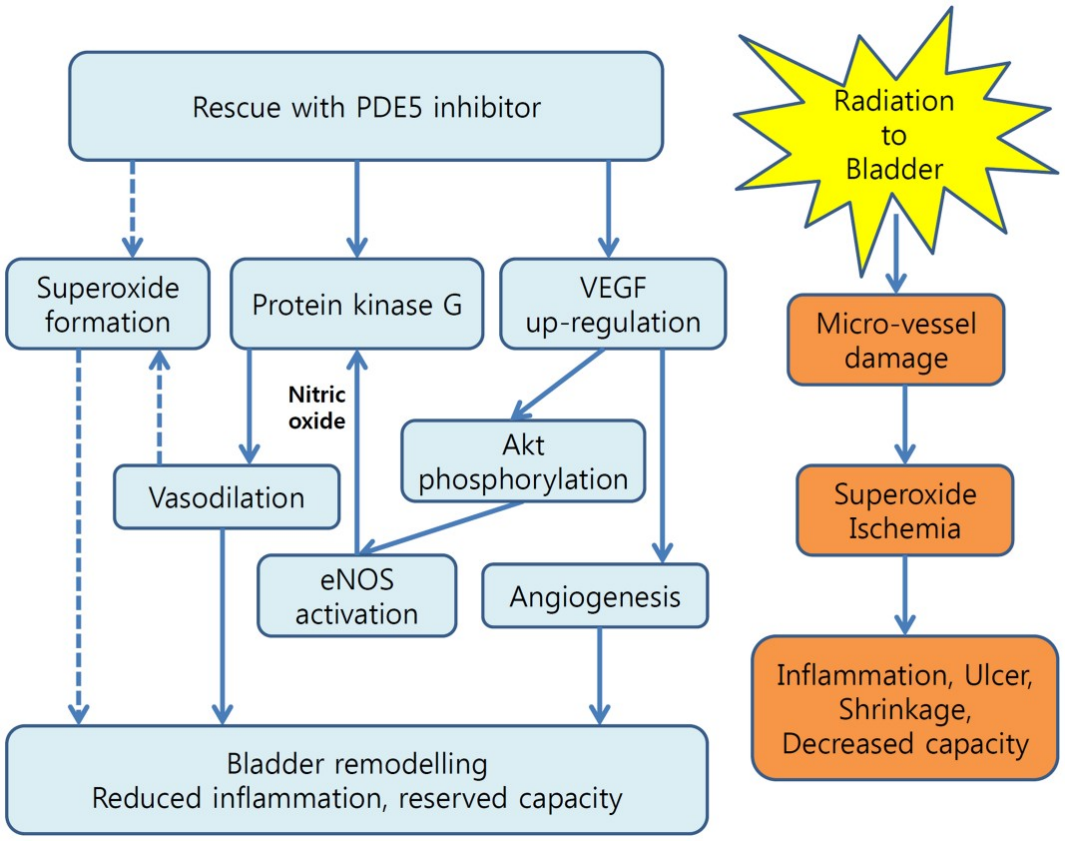
Proposed mechanism of the effect of PDE5Is on the radiation-exposed urinary bladder. Radiation damages micro-vessels in urinary bladder which subsequently leads chronic tissue ischemia resulting in bladder dysfunction. Chronic use of phosphodiesterase type 5 inhibitors could enhance angiogenesis and facilitate vasodilation via VEGF-Akt-eNOS pathway which, in turn, could reduce tissue ischemia and may induce bladder remodelling resulting in maintenance of end-organ function. Bold arrow: positive effect; dotted arrow: negative effect.

Avoiding radiation-induced bladder toxicity is still challenging. Based on the previous literature and the present study, treatment with a PDE5I may upregulate the expression of VEGF mRNA, subsequently increasing VEGF protein expression and stimulating angiogenesis. Increased VEGF secretion in response to PDE5I treatment may increase phosphorylation of Akt, which may in turn upregulate eNOS expression, resulting in the release of NO from the endothelium and an acceleration of vasodilation though PRKG activation. Therefore, PDE5I treatment may elicit bladder remodelling after radiation exposure through angiogenesis, vasodilation, and reductions in superoxide levels, finally preserving bladder function (Fig 6).

## Supporting information

S1 Table Oligonucleotide primer pairs used for RT-PCR amplification. (DOC)

S1 Raw images Original RT-PCR and western blot data. There were 4 outliers (red arrows: sample number #5 of group 2 for PDE and PRKG, and sample number #5 of group 1 and 3 for NOX2). One error (red asterisk: missing data) was found in sample number #8 of group 2 for p-Akt. Akt: protein kinase B, eNOS: Endothelial Nitric Oxide Synthase, NOX2: NADPH oxidase 2, PDE5I: Phosphodiesterase type 5 inhibitor, PRKG: cGMP dependent protein kinase, VEGF: Vascular endothelial growth factor.
(PDF)

## References

[1] Liberman D, Mehus B, Elliott SP. Urinary adverse effects of pelvic radiotherapy. Transl Androl Urol. 2014;3(2):186–95. Epub 2014/06/01. 10.3978/j.issn.2223-4683.2014.04.01 26813159 PMC4708162

[2] Lukka H, Hayter C, Julian JA, Warde P, Morris WJ, Gospodarowicz M, et al Randomized trial comparing two fractionation schedules for patients with localized prostate cancer. J Clin Oncol. 2005;23(25):6132–8. Epub 2005/09/02. 10.1200/JCO.2005.06.153 16135479

[3] Pollack A, Walker G, Horwitz EM, Price R, Feigenberg S, Konski AA, et al Randomized trial of hypofractionated external-beam radiotherapy for prostate cancer. J Clin Oncol. 2013;31(31):3860–8. Epub 2013/10/09. 10.1200/JCO.2013.51.1972 24101042 PMC3805927

[4] Lawton CA, Won M, Pilepich MV, Asbell SO, Shipley WU, Hanks GE, et al Long-term treatment sequelae following external beam irradiation for adenocarcinoma of the prostate: analysis of RTOG studies 7506 and 7706. Int J Radiat Oncol Biol Phys. 1991;21(4):935–9. Epub 1991/09/01. 10.1016/0360-3016(91)90732-j 1917622

[5] Zietman AL, DeSilvio ML, Slater JD, Rossi CJ Jr., Miller DW, Adams JA, et al Comparison of conventional-dose vs high-dose conformal radiation therapy in clinically localized adenocarcinoma of the prostate: a randomized controlled trial. JAMA. 2005;294(10):1233–9. Epub 2005/09/15. 10.1001/jama.294.10.1233 16160131

[6] Pascoe C, Duncan C, Lamb BW, Davis NF, Lynch TH, Murphy DG, et al Current management of radiation cystitis: a review and practical guide to clinical management. BJU Int. 2019;123(4):585–94. Epub 2018/08/17. 10.1111/bju.14516 30113758

[7] Pyriochou A, Zhou Z, Koika V, Petrou C, Cordopatis P, Sessa WC, et al The phosphodiesterase 5 inhibitor sildenafil stimulates angiogenesis through a protein kinase G/MAPK pathway. J Cell Physiol. 2007;211(1):197–204. Epub 2007/01/18. 10.1002/jcp.20929 17226792

[8] Nomiya M, Burmeister DM, Sawada N, Campeau L, Zarifpour M, Keys T, et al Prophylactic effect of tadalafil on bladder function in a rat model of chronic bladder ischemia. J Urol. 2013;189(2):754–61. Epub 2012/09/18. 10.1016/j.juro.2012.07.141 22982422

[9] Kilkenny C, Browne WJ, Cuthill IC, Emerson M, Altman DG. Improving bioscience research reporting: the ARRIVE guidelines for reporting animal research. PLoS Biol. 2010;8(6):e1000412 Epub 2010/07/09. 10.1371/journal.pbio.1000412 20613859 PMC2893951

[10] Zelefsky MJ, Pei X, Chou JF, Schechter M, Kollmeier M, Cox B, et al Dose escalation for prostate cancer radiotherapy: predictors of long-term biochemical tumor control and distant metastases-free survival outcomes. Eur Urol. 2011;60(6):1133–9. Epub 2011/09/06. 10.1016/j.eururo.2011.08.029 21889832 PMC4037155

[11] Fowler JF. The radiobiology of prostate cancer including new aspects of fractionated radiotherapy. Acta Oncol. 2005;44(3):265–76. Epub 2005/08/04. 10.1080/02841860410002824 16076699

[12] Yamashita S, Katsumata O. Heat-Induced Antigen Retrieval in Immunohistochemistry: Mechanisms and Applications. Methods Mol Biol. 2017;1560:147–61. Epub 2017/02/06. 10.1007/978-1-4939-6788-9_10 28155151

[13] Huh JS, Chung BH, Hong CH, Ryu JK, Kim JH, Han WK, et al The effects of testosterone replacement on penile structure and erectile function after long-term castration in adult male rats. Int J Impot Res. 2018;30(3):122–8. Epub 2018/05/05. 10.1038/s41443-017-0010-6 29725076

[14] Festing MF. Design and statistical methods in studies using animal models of development. ILAR J. 2006;47(1):5–14. Epub 2006/01/05. 10.1093/ilar.47.1.5 16391426

[15] Morelli A, Filippi S, Mancina R, Luconi M, Vignozzi L, Marini M, et al Androgens regulate phosphodiesterase type 5 expression and functional activity in corpora cavernosa. Endocrinology. 2004;145(5):2253–63. Epub 2004/02/07. 10.1210/en.2003-1699 14764637

[16] Lue TF. Erectile dysfunction. N Engl J Med. 2000;342(24):1802–13. Epub 2000/06/15. 10.1056/NEJM200006153422407 10853004

[17] Kukreja RC, Salloum FN, Das A, Koka S, Ockaili RA, Xi L. Emerging new uses of phosphodiesterase-5 inhibitors in cardiovascular diseases. Exp Clin Cardiol. 2011;16(4):e30–5. Epub 2011/12/02. 22131856 PMC3206106

[18] Salloum FN, Ockaili RA, Wittkamp M, Marwaha VR, Kukreja RC. Vardenafil: a novel type 5 phosphodiesterase inhibitor reduces myocardial infarct size following ischemia/reperfusion injury via opening of mitochondrial K(ATP) channels in rabbits. J Mol Cell Cardiol. 2006;40(3):405–11. Epub 2006/02/17. 10.1016/j.yjmcc.2005.10.002 16480739

[19] Wang X, Fisher PW, Xi L, Kukreja RC. Essential role of mitochondrial Ca2+-activated and ATP-sensitive K+ channels in sildenafil-induced late cardioprotection. J Mol Cell Cardiol. 2008;44(1):105–13. Epub 2007/11/21. 10.1016/j.yjmcc.2007.10.006 18021798

[20] Sahara M, Sata M, Morita T, Nakajima T, Hirata Y, Nagai R. A phosphodiesterase-5 inhibitor vardenafil enhances angiogenesis through a protein kinase G-dependent hypoxia-inducible factor-1/vascular endothelial growth factor pathway. Arterioscler Thromb Vasc Biol. 2010;30(7):1315–24. Epub 2010/04/24. 10.1161/ATVBAHA.109.201327 20413734

[21] Barnett GC, West CM, Dunning AM, Elliott RM, Coles CE, Pharoah PD, et al Normal tissue reactions to radiotherapy: towards tailoring treatment dose by genotype. Nat Rev Cancer. 2009;9(2):134–42. Epub 2009/01/17. 10.1038/nrc2587 19148183 PMC2670578

[22] Kimura M, Rabbani ZN, Zodda AR, Yan H, Jackson IL, Polascik TJ, et al Role of oxidative stress in a rat model of radiation-induced erectile dysfunction. J Sex Med. 2012;9(6):1535–49. Epub 2012/04/12. 10.1111/j.1743-6109.2012.02716.x 22489731

[23] Carmeliet P, Ng YS, Nuyens D, Theilmeier G, Brusselmans K, Cornelissen I, et al Impaired myocardial angiogenesis and ischemic cardiomyopathy in mice lacking the vascular endothelial growth factor isoforms VEGF164 and VEGF188. Nat Med. 1999;5(5):495–502. Epub 1999/05/06. 10.1038/8379 10229225

[24] Mattot V, Moons L, Lupu F, Chernavvsky D, Gomez RA, Collen D, et al Loss of the VEGF(164) and VEGF(188) isoforms impairs postnatal glomerular angiogenesis and renal arteriogenesis in mice. J Am Soc Nephrol. 2002;13(6):1548–60. Epub 2002/06/01. 10.1097/01.asn.0000013925.19218.7b 12039984

[25] Cheppudira BP, Girard BM, Malley SE, Schutz KC, May V, Vizzard MA. Upregulation of vascular endothelial growth factor isoform VEGF-164 and receptors (VEGFR-2, Npn-1, and Npn-2) in rats with cyclophosphamide-induced cystitis. Am J Physiol Renal Physiol. 2008;295(3):F826–36. Epub 2008/07/18. 10.1152/ajprenal.90305.2008 18632792 PMC2536878

[26] Lai HH, Shen B, Vijairania P, Zhang X, Vogt SK, Gereau RWt. Anti-vascular endothelial growth factor treatment decreases bladder pain in cyclophosphamide cystitis: a Multidisciplinary Approach to the Study of Chronic Pelvic Pain (MAPP) Research Network animal model study. BJU Int. 2017;120(4):576–83. Epub 2017/06/06. 10.1111/bju.13924 28581681 PMC5716917

[27] Sokolakis I, Dimitriadis F, Psalla D, Karakiulakis G, Kalyvianakis D, Hatzichristou D. Effects of low-intensity shock wave therapy (LiST) on the erectile tissue of naturally aged rats. Int J Impot Res. 2019;31(3):162–9. Epub 2018/08/19. 10.1038/s41443-018-0064-0 30120384

[28] Khan MA, Thompson CS, Mumtaz FH, Mikhailidis DP, Morgan RJ, Bruckdorfer RK, et al The effect of nitric oxide and peroxynitrite on rabbit cavernosal smooth muscle relaxation. World J Urol. 2001;19(3):220–4. Epub 2001/07/27. 10.1007/s003450000162 11469612

[29] Manning BD, Cantley LC. AKT/PKB signaling: navigating downstream. Cell. 2007;129(7):1261–74. Epub 2007/07/03. 10.1016/j.cell.2007.06.009 17604717 PMC2756685

