# Supplementary material for: A role for phosphodiesterase type 5 inhibitors in remodelling the urinary bladder after radiation exposure
Source: PLoS One. 2020 Nov 9;15(11):e0242006. doi: 10.1371/journal.pone.0242006 (PMC7652354; doi:10.1371/journal.pone.0242006)
Supplement: S1 Table — (DOC) [file pone.0242006.s001.doc]

S1 Table. Oligonucleotide primer pairs used for RT-PCR amplification

| Target | NCBI accession no. | Sequence(5'→3') | Size(bp) | Temperature  (℃) |
| --- | --- | --- | --- | --- |
| GAPDH | NG_028301 | F: ACGGGAAACCCATCACCATC | 311 | 57 |
|  |  | R: CCCTTCCACGATGCCAAAGT |  |  |
| VEGFa | NM_001287108.1 | F:GCACATAGGAGAGATGAGCTTCC | 231 | 58 |
|  |  | R:CACCGCCTTGGCTTGTCACAT |  |  |
| PDE5 | NM_133584.1 | F:CTGTCTGATCTGGAAACAGC | 251 | 60 |
|  |  | R:GCAATCAGCAATGCAAGCGT |  |  |
| eNOS | XM_006235872 | F: TCCAGAGCATACCCGCACTTC | 393 | 58 |
|  |  | R: GTCCAGACGCACCAGGATTG |  |  |
