# Supplementary material for: A role for phosphodiesterase type 5 inhibitors in remodelling the urinary bladder after radiation exposure
Source: PLoS One. 2020 Nov 9;15(11):e0242006. doi: 10.1371/journal.pone.0242006 (PMC7652354; doi:10.1371/journal.pone.0242006)
Supplement: S1 Raw images — There were 4 outliers (red arrows: sample number #5 of group 2 for PDE and PRKG, and sample number #5 of group 1 and 3 for NOX2). One error (red asterisk: missing data) was found in sample number #8 of group 2 for p-Akt. Akt: protein kinase B, eNOS: Endothelial Nitric Oxide Synthase, NOX2: NADPH oxidase 2, PDE5I: Phosphodiesterase type 5 inhibitor, PRKG: cGMP dependent protein kinase, VEGF: Vascular endothelial growth factor. (PDF) [file pone.0242006.s002.pdf]

PDE mRNA expression

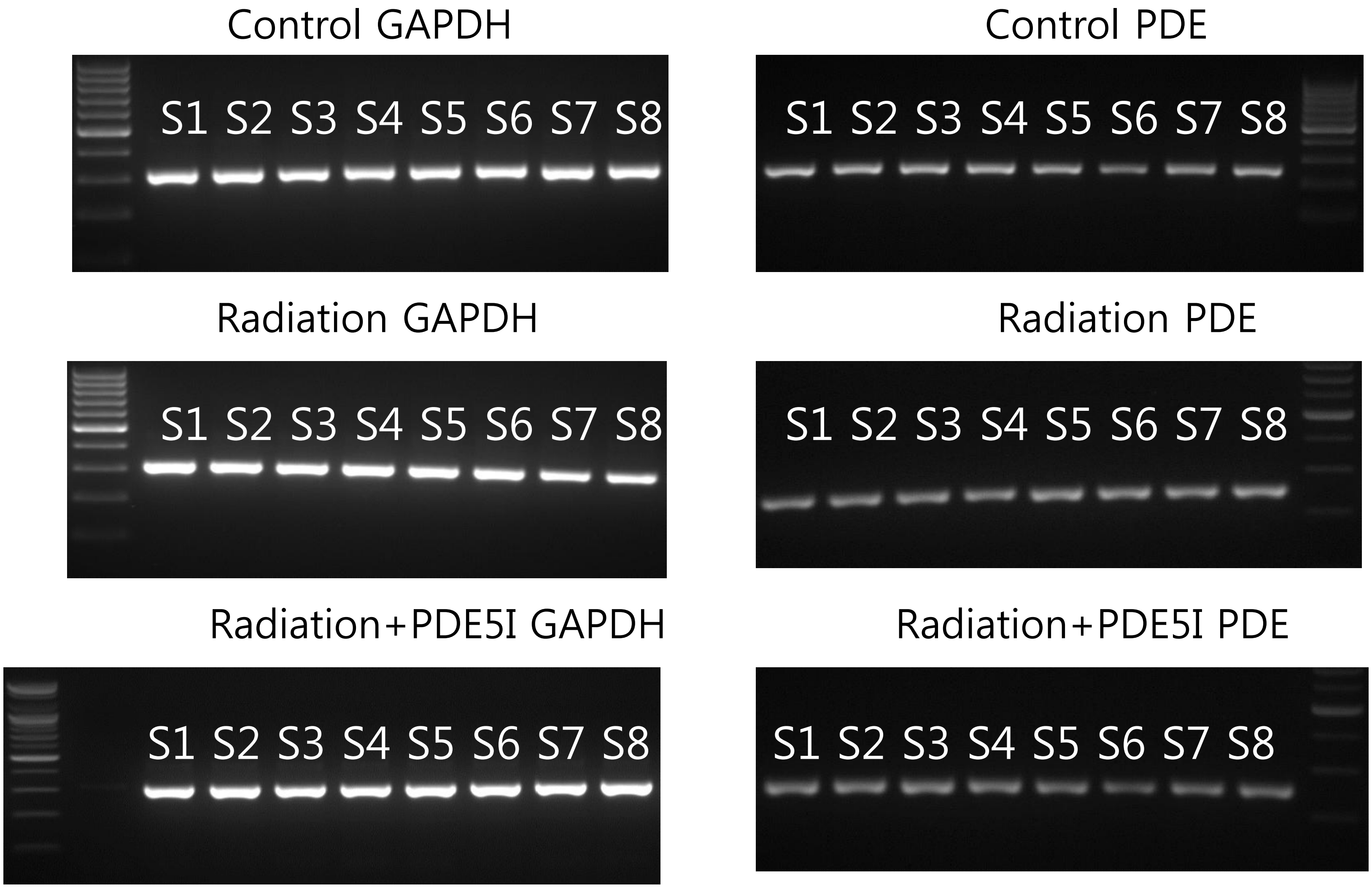

VEGF mRNA expression

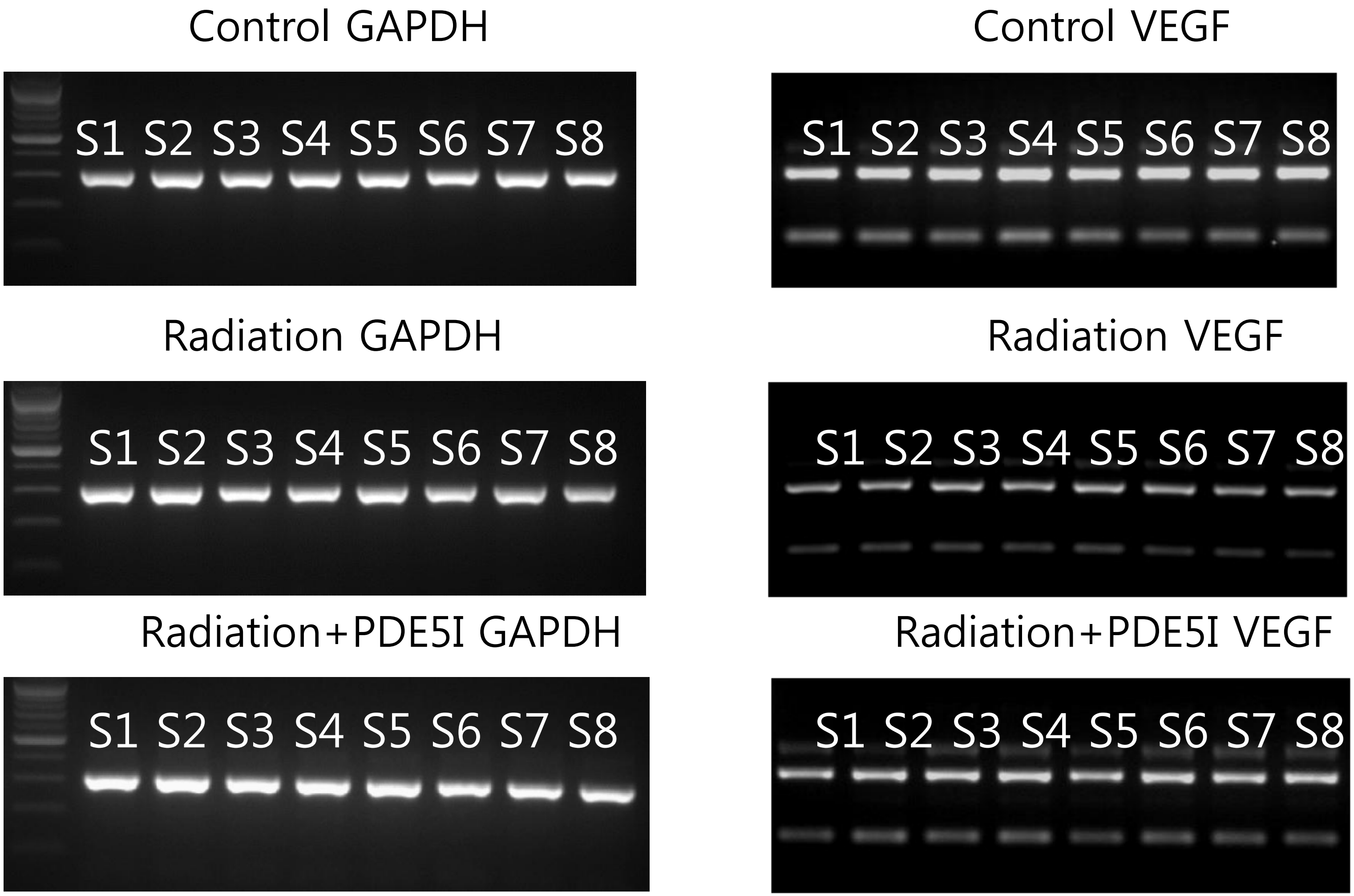

eNOS mRNA expression

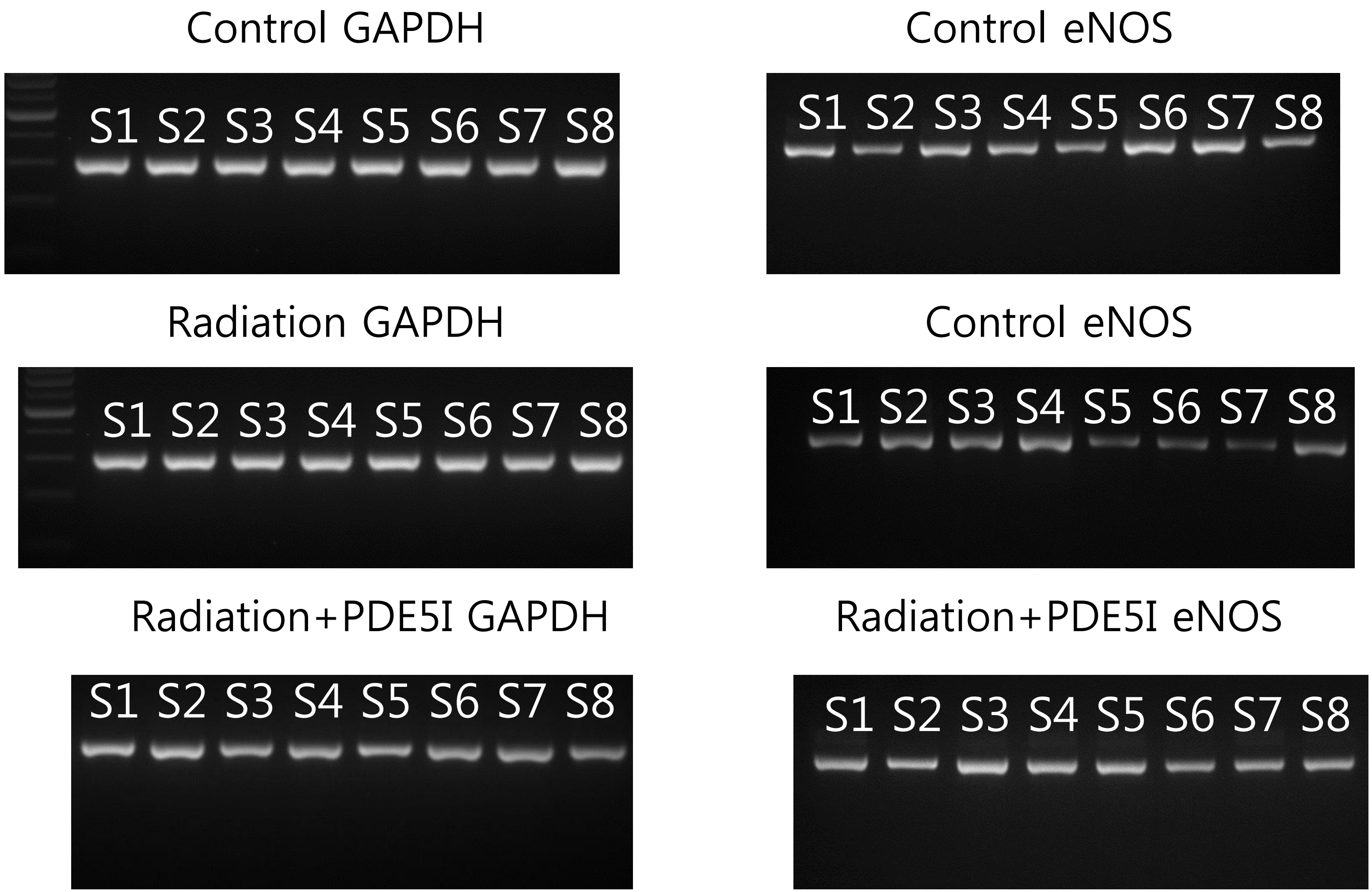

beta-Actin expression

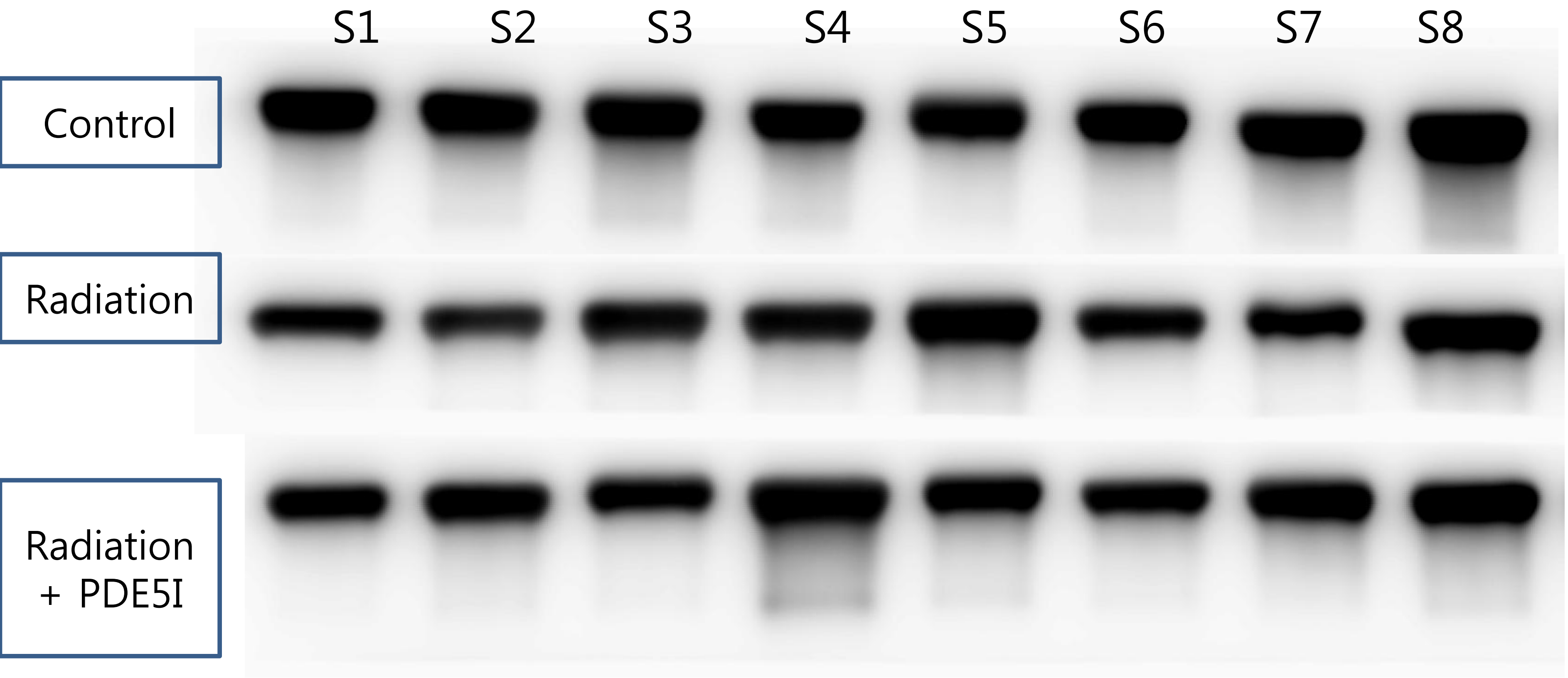

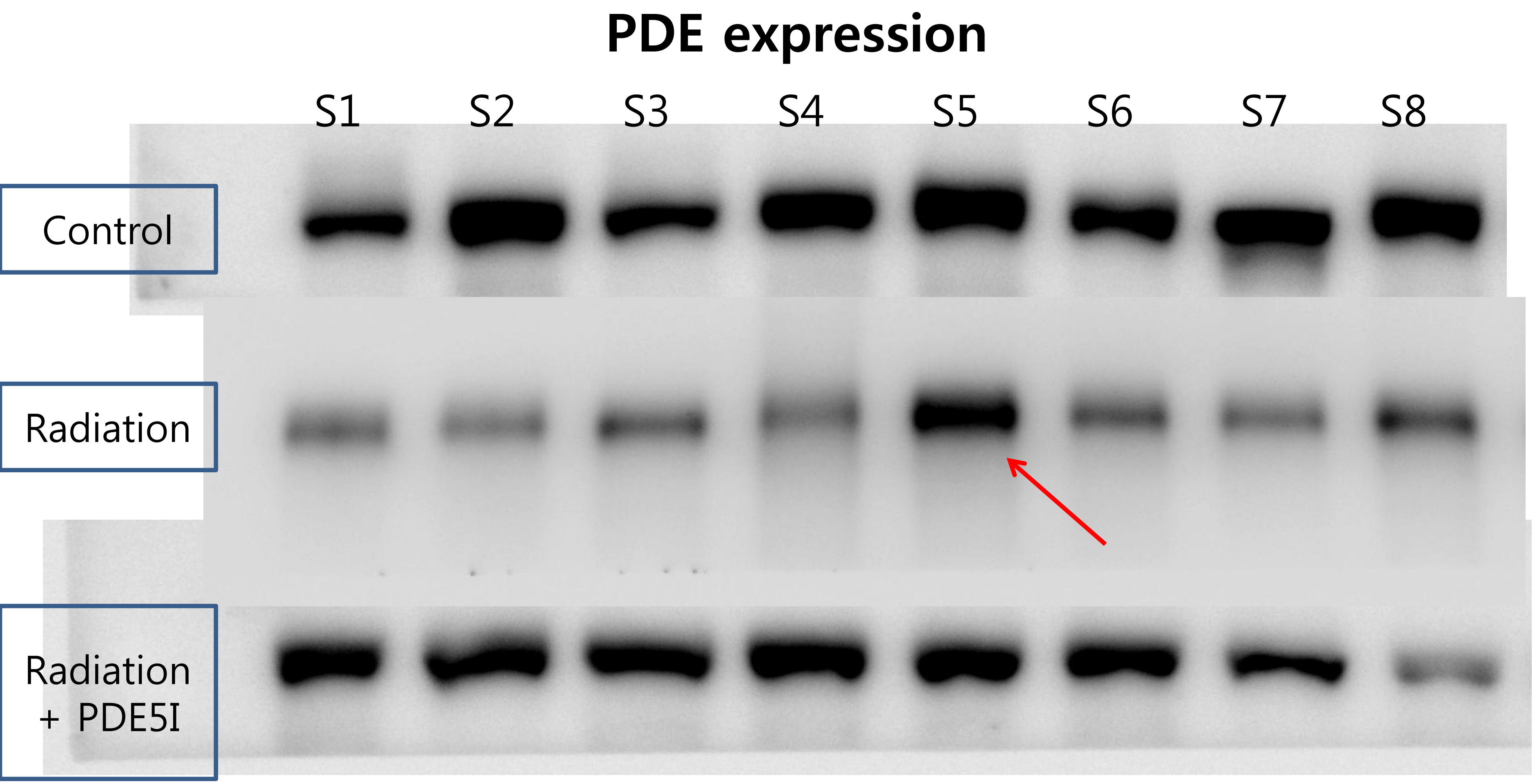
PDE expression
S1
S2
S3
S4
S5
S6
S7
S8
Control
Radiation
Radiation + PDE5I

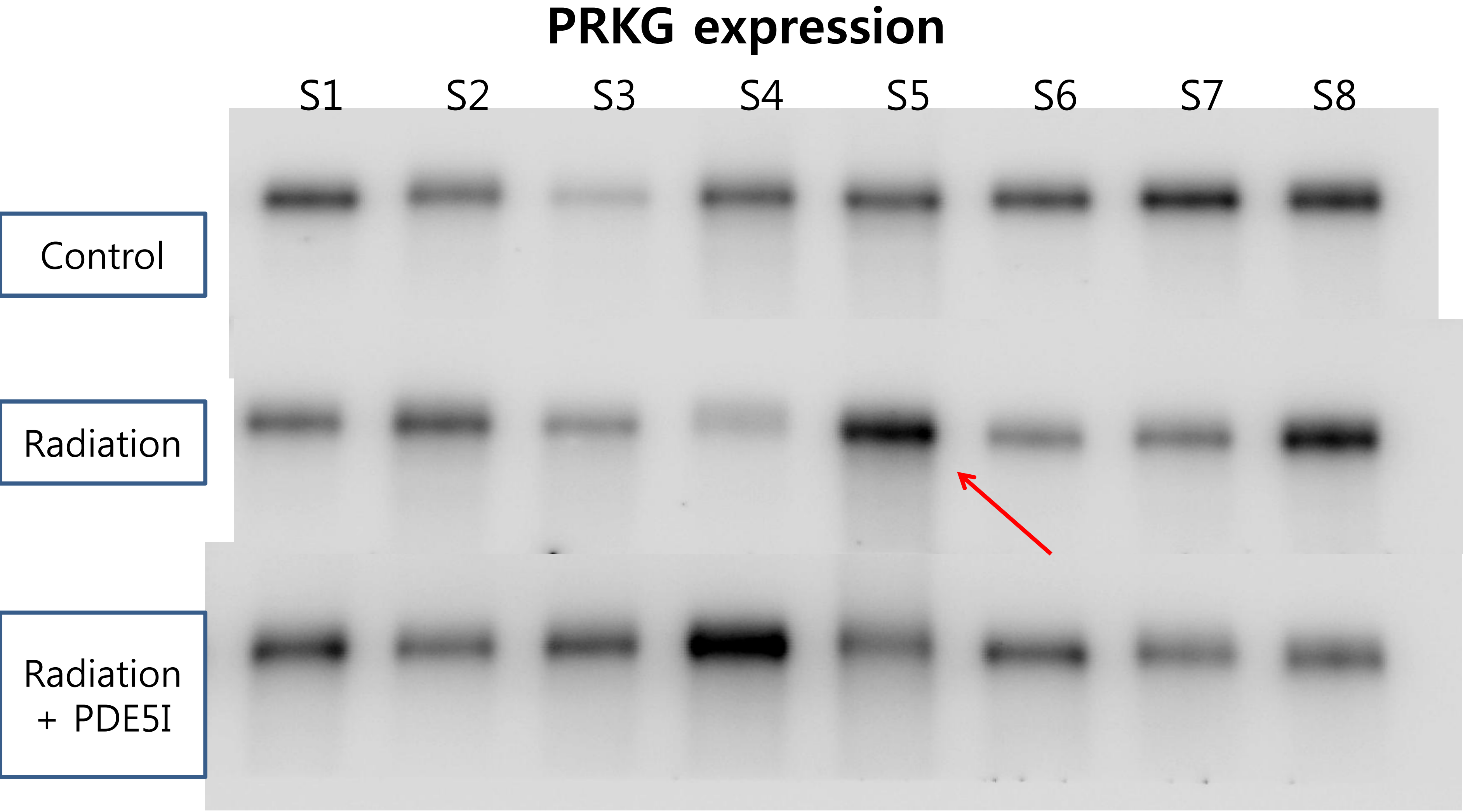
PRKG expression
S1
S2
S3
S4
S5
S6
S7
S8
Control
Radiation
Radiation + PDE5I

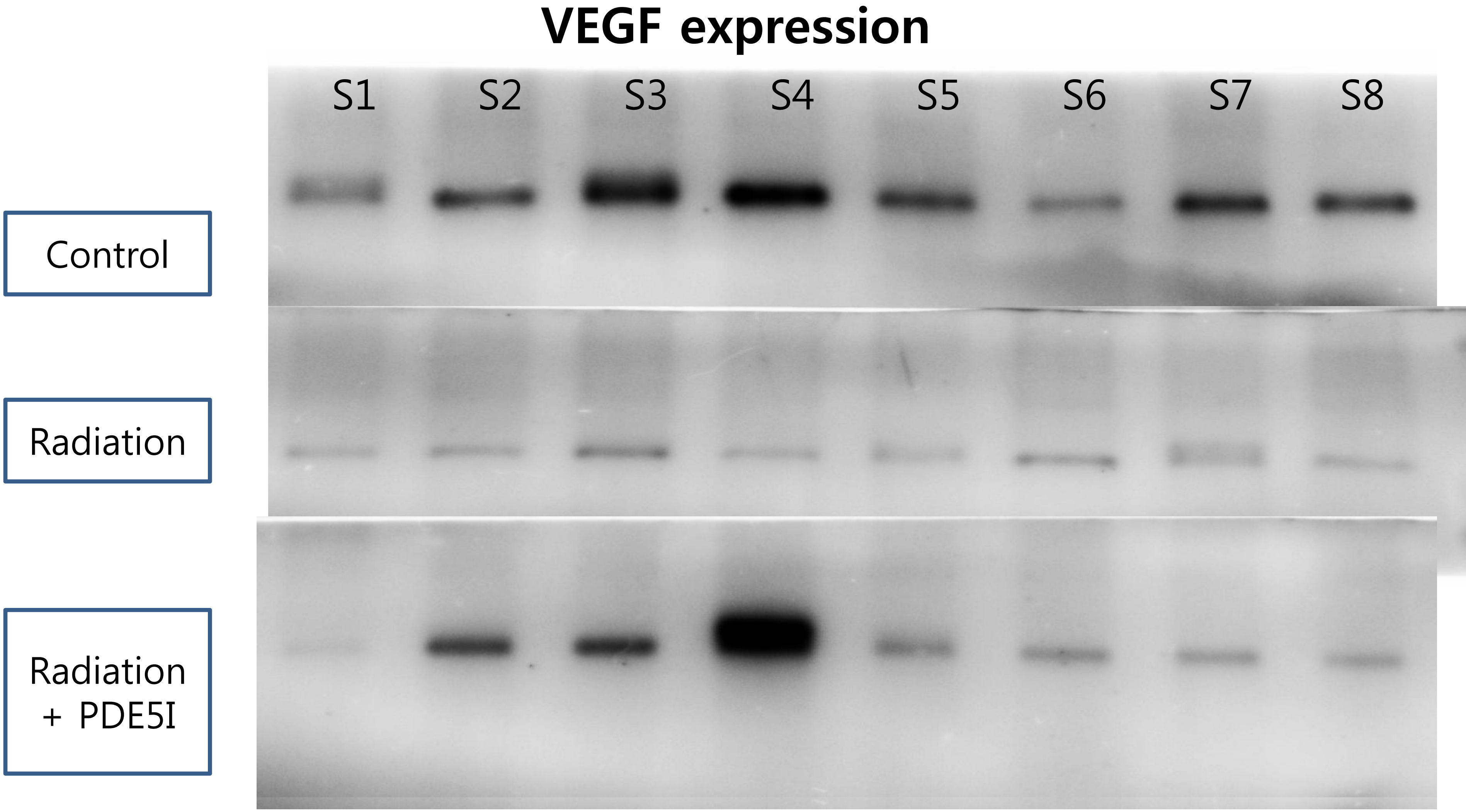
VEGF expression
S1
S2
S3
S4
S5
S6
S7
S8
Control
Radiation
Radiation + PDE5I

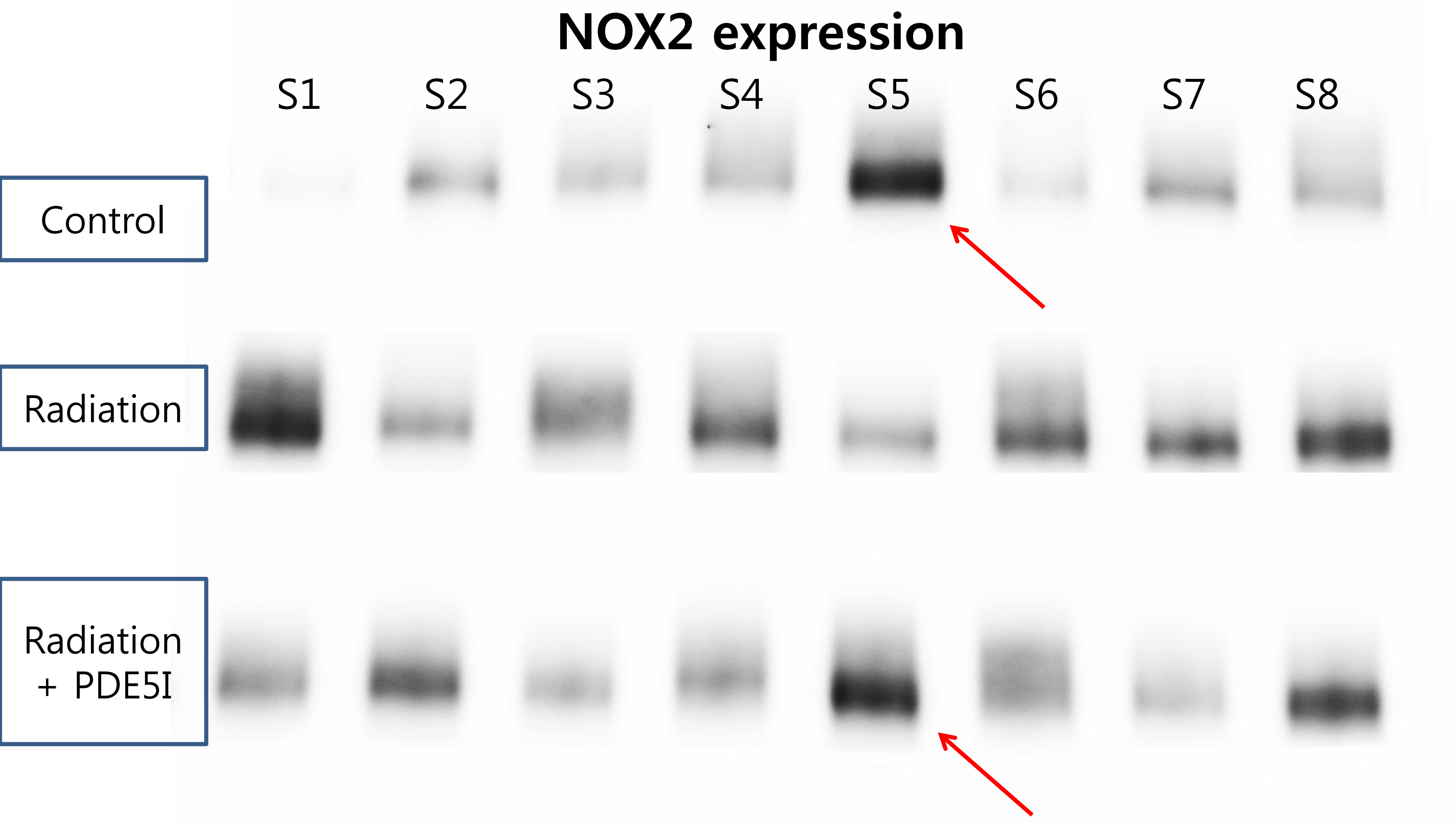

NOX2 expression
S1
S2
S3
S4
S5
S6
S7
S8
Control
Radiation
Radiation
+ PDE5I

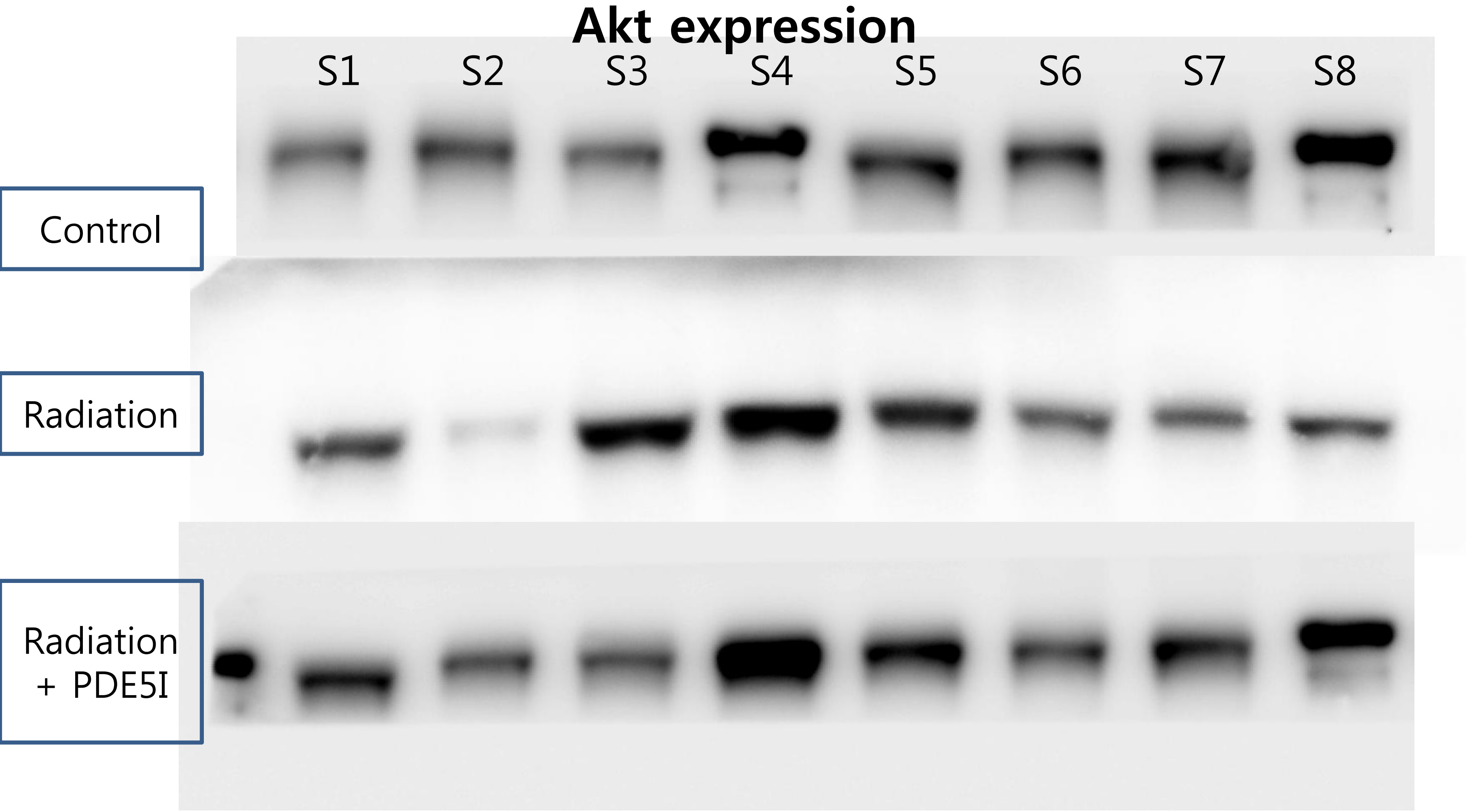
Akt expression
S1
S2
S3
S4
S5
S6
S7
S8
Control
Radiation
Radiation
+ PDE5I

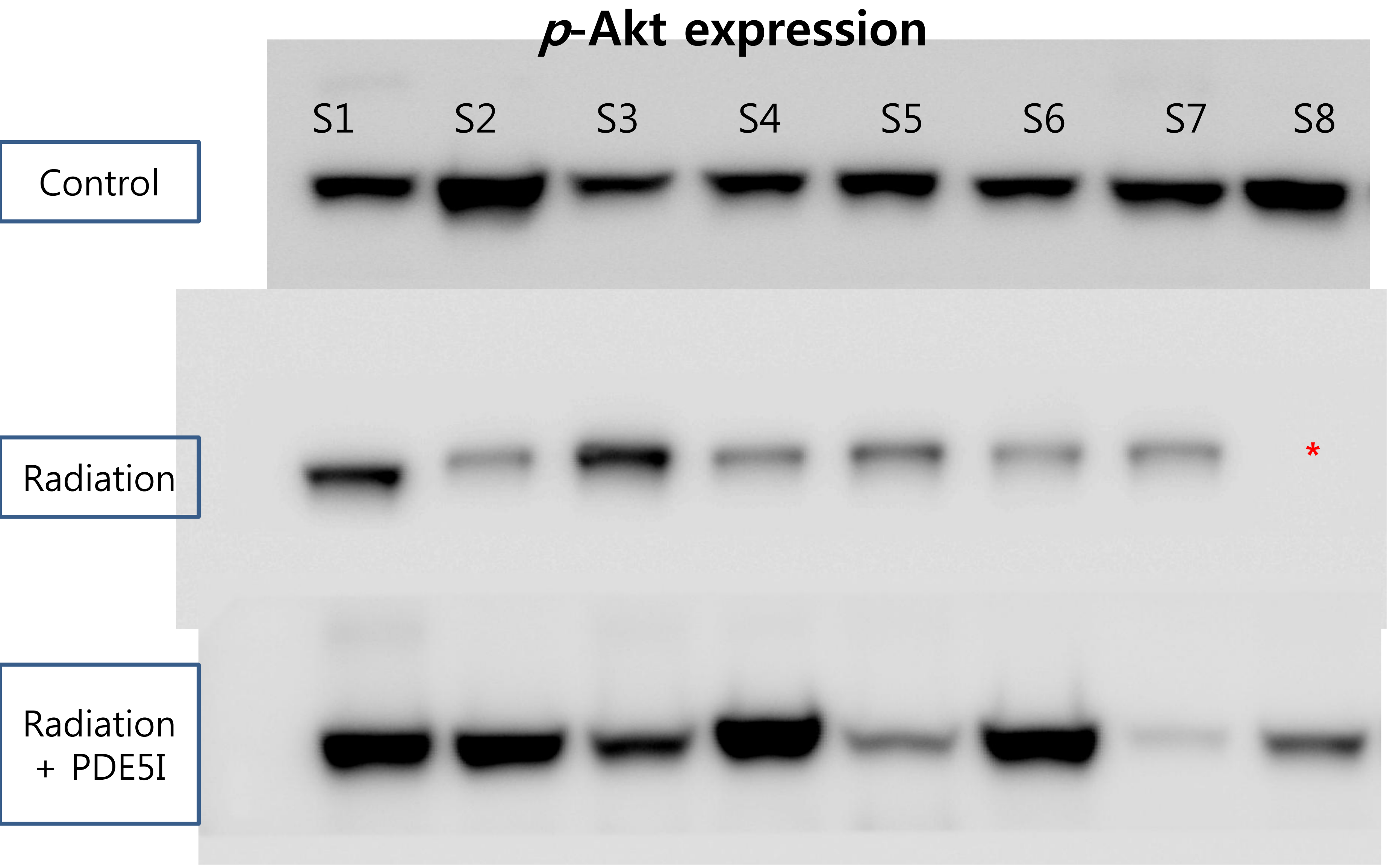

p-Akt expression
S1
S2
S3
S4
S5
S6
S7
S8
Control
Radiation
Radiation + PDE5I
*

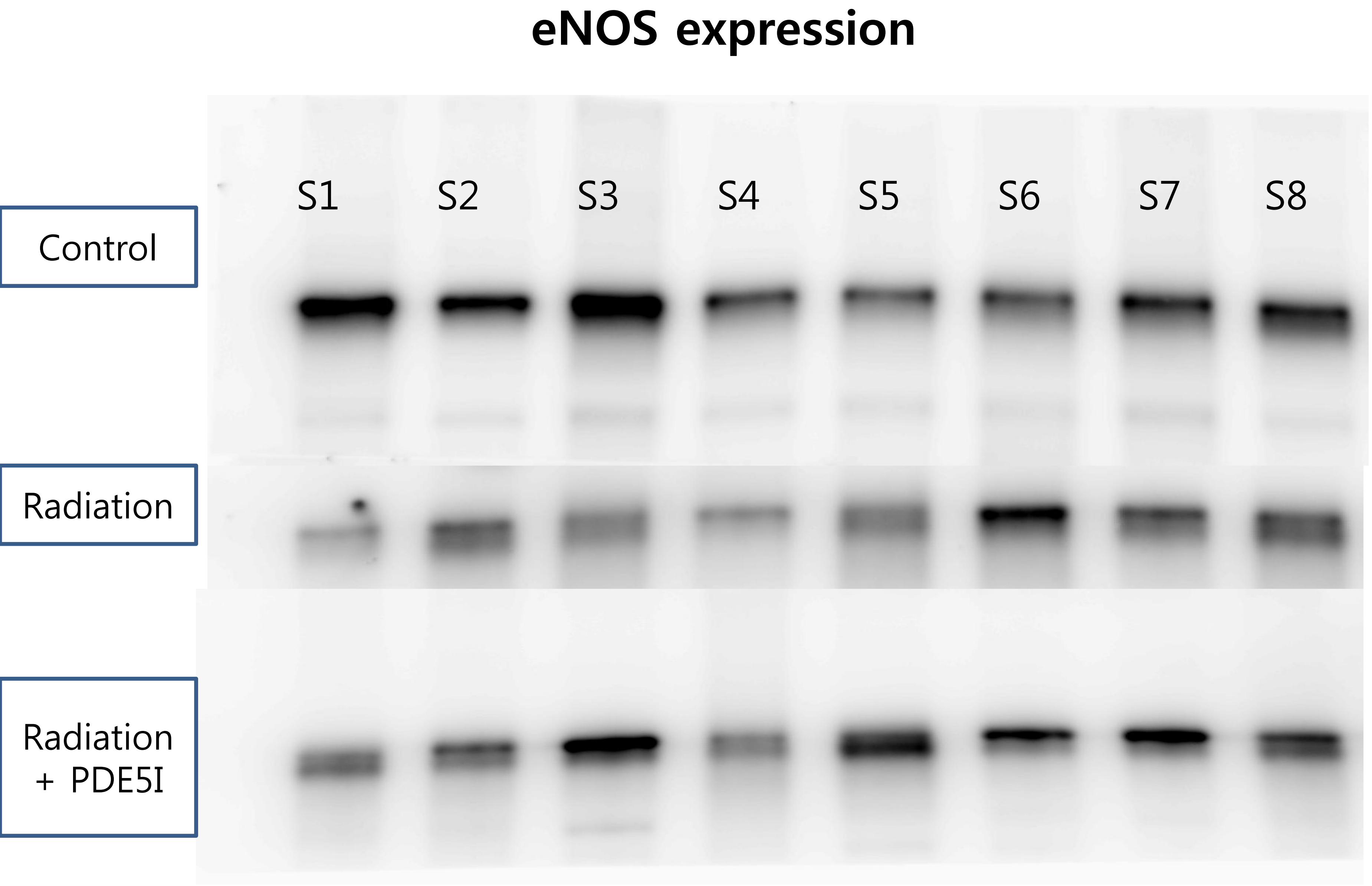
eNOS expression
S1
S2
S3
S4
S5
S6
S7
S8
Control
Radiation
Radiation + PDE5I
